# Impact of a decision-support tool on decision making at the district level in Kenya

**DOI:** 10.1186/1478-4505-11-34

**Published:** 2013-09-08

**Authors:** Tara Nutley, Sarah McNabb, Shannon Salentine

**Affiliations:** 1 MEASURE Evaluation, Futures Group, 308 West Rosemary Street, Chapel Hill, NC 27516, USA; 2 Futures Group, One Thomas Circle, NW Suite 200, Washington, DC 20005, USA; 3 MEASURE Evaluation, ICF International, 308 W. Rosemary Street, Chapel Hill, NC 26516, USA

**Keywords:** Data-informed decision making, Data dashboards, Data use, Decision-support tools, District level, Health information systems, Health systems strengthening

## Abstract

**Background:**

In many countries, the responsibility for planning and delivery of health services is devolved to the subnational level. Health programs, however, often fall short of efficient use of data to inform decisions. As a result, programs are not as effective as they can be at meeting the health needs of the populations they serve. In Kenya, a decision-support tool, the District Health Profile (DHP) tool was developed to integrate data from health programs, primarily HIV, at the district level and to enable district health management teams to review and monitor program progress for specific health issues to make informed service delivery decisions.

**Methods:**

Thirteen in-depth interviews were conducted with ten tool users and three non-users in six districts to qualitatively assess the process of implementing the tool and its effect on data-informed decision making at the district level. The factors that affected use or non-use of the tool were also investigated. Respondents were selected via convenience sample from among those that had been trained to use the DHP tool except for one user who was self-taught to use the tool. Selection criteria also included respondents from urban districts with significant resources as well as respondents from more remote, under-resourced districts.

**Results:**

Findings from the in-depth interviews suggest that among those who used it, the DHP tool had a positive effect on data analysis, review, interpretation, and sharing at the district level. The automated function of the tool allowed for faster data sharing and immediate observation of trends that facilitated data-informed decision making. All respondents stated that the DHP tool assisted them to better target existing services in need of improvement and to plan future services, thus positively influencing program improvement.

**Conclusions:**

This paper stresses the central role that a targeted decision-support tool can play in making data aggregation, analysis, and presentation easier and faster. The visual synthesis of data facilitates the use of information in health decision making at the district level of a health system and promotes program improvement. The experience in Kenya can be applied to other countries that face challenges making district-level, data-informed decisions with data from fragmented information systems.

## Background

Quality data are the foundation to health system improvements [1-3]; however, health programs frequently fall short of efficient use of data to inform decisions [4]. Too often, data linger in reports and databases, and are not sufficiently used to inform program development and improvement, policy development, strategic planning, or advocacy. Part of the reason for the breakdown in the process is that health information systems (HIS)^a^ are fragmented, complex, and do not fully respond to information needs [4-10]. As a result, decision makers are often unable to access the data they need in a timely manner to inform their upcoming decisions.

A number of decision-support tools for use in the health sector have been developed to address some of the barriers to data-informed decision making. Decision-support tools synthesize and display data to inform priority decisions. Data dashboards, health summary bulletins, health status report cards, and color-coded data presentation techniques are all examples of this type of tool [11]. Decision-support tools aim to improve understanding and use of data to support decision making by improving access to data, linking key data sources, helping data users navigate large data sets, and providing a variety of options for synthesizing and displaying data according to need. Decision-support tools have also been found to support evidenced-based decision making by improving data quality and availability [12], and by providing tools for analysis and interpretation of data in relation to national-, district-, or local-level information needs [5,11,13-18]; the World Health Organization (WHO) lists them as a key attribute of a national health strategy [5].

This paper focuses on the use of electronic decision-support tools, specifically software that routinely organizes data collected to support planning, budgeting, or other health priority-setting activities [19]. Not included in this definition are decision-support tools that are products of one-time data collection and/or do not have an auto update function. There are many different types of electronic decision-support tools, ranging from comprehensive tools that address multiple health programs to specific tools that address just one health program or specific elements of a health program. Comprehensive decision-support tools can serve to quickly integrate and streamline data from multiple sources including Demographic and Health Survey datasets, census statistics, and routinely collected data (project- or facility-specific) [4,20,21]. These types of systems commonly have a data dashboard function that allows data users and decision makers to easily and quickly view data on multiple health programs at different levels of the health system (national, regional, district, health facility) at regular intervals. The dashboard function visually synthesizes data related to certain health indicators or service programs [11,12,22]. By giving data users the tools to select specific data and quickly view them together, decision makers can tailor large databases to their information needs. Decision-support tools can also be more specific and are often designed to support targeted information needs including individual patient tracking, case management, program operations, and capacity building at the program or health facility level; they can also be used as supplements to national health information systems.

While there are many types and purposes of electronic decision-support tools, we found no published articles in the peer-reviewed literature of their effect on decision making. Papers discussing the need for improved data-informed, district-level decision making are found [23-26], but there were no examples of the implementation of electronic decision-support tools, as defined in this paper, to meet that need. The need for decision-support tools at the district level is particularly great, and the evaluation of their effect on improvements in data-informed decision making is warranted because of the current reality of the decentralization of health services as a key element of health system reform [25,26]. Many countries make decisions about how best to provide services to their communities without regular access to the necessary data to inform these decisions [25]. The aim of this paper is to provide an example of the development and application of a decision-support tool at the district level in Kenya and its effect on data-informed decision making.

### Health information systems in Kenya

Since 2003, various studies have been undertaken in Kenya to assess the HIS and efforts have been implemented to improve the system. However, in 2008, after years of investment in their HIS, the Government of Kenya was still struggling to access and use quality and timely data to inform health decision making. This was particularly evident at sub-national levels where the lack of a system for improving data access, synthesis, communication, and interpretation was inhibiting districts to make decisions about key service delivery issues [27].

An assessment of the health management information system conducted between 2006 and 2007 [28] described the existing routine HIS as fragmented and vertical with stand-alone systems at the national level. The paper-based, vertical systems resulted in data being “largely unavailable for effective planning, monitoring, and evaluation at all levels [of the health system]” [28]. The National Health Information Strategy, developed in 2009 [27], identified additional gaps related to insufficient use of data in decision making. The health information strategy addressed these needs by calling for the elimination of the vertical nature of the routine HIS and the integration of existing data sources into one data warehouse. The District Health Information System (DHIS), an open-source, web-based health management information system was identified as a key solution in the health information strategy and was implemented beginning in 2010. It was envisioned that introduction of the DHIS would improve data use at all levels of the health system.

The APHIA II Evaluation Project, funded by the U.S. Agency for International Development (USAID), partnered with the Government of Kenya from 2008 to 2011 to improve the functioning of the Kenyan Ministry of Health’s (MOH) information systems and build capacity to support data-informed decision making; the project complemented ongoing work to strengthen the national HIS mentioned above. In addition to working at the national level, APHIA II Evaluation prioritized work at the district level to improve the capacity of district health professionals to report and use district-level information.

Through a consultative process with country stakeholders that began in February 2009, APHIA II Evaluation, in collaboration with the MOH and National AIDS Control Council (NACC), developed the District Health Profile (DHP) tool. The tool was the beginning of a solution to integrate data from various health programs, primarily HIV-related, at the district level and enable district health management teams (DHMTs) to comprehensively review those data to make informed service delivery decisions. While the DHIS was the primary vehicle to serve this purpose, it was predicted that the DHIS would not be fully operational in all districts for up to one to two years. The possible overlap of the two tools was noted, but the value of a stop-gap tool was recognized. Moreover, the DHP tool was designed to meet specific and targeted information needs while the DHIS has a much broader scope. The DHP tool specifically addresses 11 priority health questions which include:

• Are HIV positive individuals who are eligible for treatment receiving treatment?

• Are HIV positive individuals receiving both facility & community - based support?

• Are testing & counseling services reaching the populations in need?

• Are pregnant women who seek ANC services being tested & HIV+ women receiving prophylaxis?

• Are pregnant women who seek maternity services being tested & HIV+ women receiving prophylaxis?

• Are HIV+ women receiving preventive prophylaxis for their babies?

• Are OVC (orphans and vulnerable children) services reaching the populations in need?

• Are pregnant women accessing maternity services?

• How many family planning methods have been distributed this quarter?

• Are family planning services reaching new clients?

• How well are facilities and partners reporting to the HIS?

In addition, the DHP tool creates indicators and provides analysis tools to answer the questions. Regular review and discussion of the data represented in the DHP tool was intended to alert program managers to potential problems in the service delivery areas included in the tool. It was decided that the DHP tool would first be pre-tested in a small number of districts. Once the tool was piloted and refined, the system would be rolled out at the provincial level.

### Decision-support: the DHP tool

The DHP tool responds to ten health questions and one data quality question deemed priority by key stakeholders. It links to existing MOH monthly data aggregation in Microsoft Excel spreadsheets and aggregates and analyzes them with other relevant data on a quarterly basis. The tool produces reports that contain auto-generated graphs, by district, in response to the 11 questions. Accompanying the tool is an explanation of how each graph is constructed, the data sources for each graph, and guidance for how to apply the DHP tool to facilitate district-level programmatic decision making.

**Figure 1:**
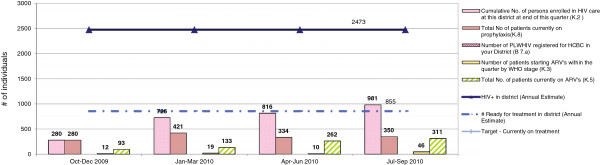
Sample DHP tool output graph.

The tool also imports standardized denominator data. These data came from the annual operational plan, a process that is undertaken each year by the MOH to budget and plan for the upcoming year. Although these data were previously available, they were never connected to routine service delivery data. In addition to providing estimates by district on the population in need of services, program targets can also be entered into the tool. The inclusion of the denominator data and targets allows users to review their service delivery data against their district targets so that they can assess their quarterly progress towards these targets (Figure 1).

The DHP tool also has features intended to address poor data quality. Data aggregation is done with minimal data entry as the tool links to existing MOH Excel data compilation spreadsheets. Users select their district from a dropdown menu and graphs that respond to the 11 questions are auto-generated, thereby eliminating manual steps involved in data aggregation, analysis, and presentation. The automatic function of the tool not only enables users to generate complex graphs effortlessly, but also reduces errors in these steps.

The DHP tool was purposively created using available and familiar technology: Microsoft Excel in an Office 2003 environment. The file used VBA (Visual Basic for Applications) to perform calculations and insert data into the expected locations in order to generate the charts. Inno Setup (freely available) was used to package all the files together and create an installable application. A users’ guide was developed to orient users on how to install and set up the tool, configure macros, enter data, run reports, print reports, and troubleshoot. The guide also explains the rationale for the tool, the data sources, the calculations behind the graphs, and how to interpret the graphs in a programmatic context.

In May 2009, 25 MOH master trainers from the national and district levels were trained to use the tool and encouraged to pilot the tool in their work settings. The tool was distributed to users via flash drive at these events and to subsequent users via email. After seven weeks, the group reconvened to provide suggestions for improving the tool; tool developers incorporated these suggestions into a second version of the tool.

### National roll-out of the DHP tool

The National AIDS and STD Control Program (NASCOP) launched the national roll-out of the DHP tool, version 2, by training representatives from the national program and select regions as master trainers in how to use the tool. They then invited all districts in Kenya to send representatives to be trained in how to use the tool. In November 2010, a total of 30 trainers and 489 district health team members from 168 districts (out of 208 total districts) in Kenya were trained [29]. Training participants included district health information and records officers (DHIROs), district medical officers of health (DMOHs), district AIDS and STI control officers (DASCOs), provincial AIDS and STI control officers, provincial health information and records officers, and representatives of NASCOP and United States government partners.

## Methods

As part of the national roll-out process, a plan was developed and implemented to assess the effect of the DHP tool on data-informed decision making. In-depth interviews were implemented at six months post-training (June 2011) with ten tool users in six districts to qualitatively document the process of implementing the tool and its effect on data-informed decision making at the district level. The interviews also sought to investigate the factors that affected use or non-use of the tool; thus, three individuals who were trained to use the tool but were not using it were also interviewed (Table 1). Respondents were selected via convenience sample from among those that had been trained to use the DHP tool except for one user who did not attend a formal training event but was self-taught to use the tool. Selection criteria also included respondents from urban districts with relatively significant resources as well as respondents from more remote, under-resourced districts. The proximity of the districts to Nairobi, the capital city, was also an important selection criterion. The initial sampling plan included a total of 20 interviews with an equal number of tool users and non-users as well as an equal number of data users (individuals who manage and oversee program activities, the DHMOs and DASCOs) and data producers (individuals who manage and oversee data collection, the DHIROs). However, often when the interview team arrived at the health facility to conduct the scheduled interviews, individuals who had agreed to be interviewed were not present. Due to budget limitations it was not possible to return to the sites to interview the intended sample. Table 1 shows the specific districts and individuals interviewed.

**Table 1:** In-depth interview respondents

**District**	**DHP tool users (participated in training)**	**DHP tool non-users (participated in training)**	**DHP tool users (did not participate in training)**	**Total interviews**
Athi River	DHIRO, DASCO, DMOH			3
Embakasi	DHIRO, DASCO			2
Keito	DHIRO	DMOH, DASCO		3
Kiambu East	DASCO		DHRIO	2
Meru	DHRIO	DASCO		2
Nandi East	DMOH			1
Total				13

An open-ended, semi-structured questionnaire was used by a trained qualitative expert to collect information from individual informants. Specifically, the questions asked were: how was the DHP tool used in the district? Who were the DHP tool reports shared with? How did the DHP tool affect data review, sharing, and decision making in the district? What factors facilitated the use of the DHP tool? What factors inhibited the use of the DHP tool? What were the unexpected results of using the tool? and, What were the suggested changes to the tool? Detailed notes were taken during the interviews by a trained qualitative note taker. The information collected was synthesized and then grouped under themes that responded to the assessment objectives.

## Results

### Sharing DHP tool findings

All users of the DHP tool reported using the tool to produce regular reports to share with key health officials. All respondents reported sharing results of the DHP tool at provincial meetings and at meetings of the DHMT. DHMT members include health department coordinators and the departmental heads of the district hospital or health facility. One DHRIO explained how the tool improved collaboration with colleagues by offering the possibility to discuss an issue with the DMOH instead of waiting to follow the schedule of the DHMT meeting.

All DHRIOs reported sharing reports with stakeholders beyond the DHMT as well. Recipients included health officials at facilities who contributed the data to the tool and members of supervisory, immunization and comprehensive care meetings. The DHRIOs also noted that they shared results at the national level in the form of protocol e-mails.

### Reviewing program trends and targets

All interviewed DMOHs, DHRIOs, and DASCOs who were using the tool explained how the DHP tool facilitated reporting, analysis, and the ability to track trends against targets. One DASCO reported that he could now work with the DHRIO to easily generate reports that show trends and draw conclusions about program progress. A DHRIO reported that the tool helps to review quarterly achievements during the DHMT meeting because meeting members are now able to gauge the uptake of services in the district. One DASCO explained how the DHP makes it easier to ensure that the targets are met based on what was documented at the beginning of the quarter; he also noted that this process works to improve future target setting.

The DHRIOs and DASCOs explained how they also use the tool for personal data review. Two DHRIOs reported that they generate reports routinely, whether requested or not, because the reports help them to know if they have been able to achieve the targets laid out in their work plans.

### Improving work functioning and quality

Several informants explained how the tool has improved their ability to do their jobs. For instance, one DASCO explained that the tool has made work easier for him by enabling him to quickly gather data from various reports and present those data, which in turn supported faster data sharing and immediate observation of trends. He also explained how the improved efficiency in his work had enabled him to forward reports to his superiors on time.

Users of the tool also reported how the DHP tool has improved data quality by enabling users to identify potential discrepancies in their data. One DASCO reported that the quality of data has improved now that inflated figures can be visually identified and queried. One DHRIO explained how the tool impacted his own awareness of data quality; when he was entering data on prevention of mother-to-child transmission of HIV (PMTCT) the facility report showed 900 mothers yet the DHP tool showed 700 mothers; this alerted him to check on the accuracy of what was entered.

### Data-informed decision making

All users of the DHP tool spoke to the tool’s impact on problem solving within their district’s programs. One DHIRO explained that if problems were noted in the DHP tool findings, they go back to the raw data and identify the specific facility that is not performing and identify a solution for how to address the problem. All respondents stated that the DHP tool assisted them to better target existing services in need of improvement and to plan future services, because they can now easily see data that they did not have before but needed for planning purposes. Specifically, one DASCO stated that she can now see the cumulative number of women tested (in both maternity and PMTCT programs) to understand the overall reach of the program; before, if this was done, it had to be done manually. The ease of generating the cumulative number assists her to view all PMTCT data together to see where she needs to improve service delivery relative to all services delivered. She stated that she can now identify if she needs to focus on improving the delivery of prophylaxis to women or infants or both, or if they should work to improve counseling. She also explained how it was helpful to see, at the same time, the total population of women in need of services in order to understand the district’s role in meeting the needs of the target population.

Other respondents provided specific examples of how the DHP tool influenced program improvement. One DHRIO explained how the DHP tool allowed the DHRIO to identify a new trend of maternity dropouts. This discovery prompted the DHRIO to investigate further and implement a solution to reduce dropouts. Through continued monitoring, an increase of approximately 10% of mothers delivering at the health facility was noted, ultimately helping the facility to meet maternity targets. Another respondent shared that the DHP tool showed that voluntary counseling and testing (VCT) uptake had declined drastically. Upon further investigation, it was discovered that testing kits were not available and that VCT clients were being turned away because of staff shortages. Based on these findings, the DASCO ensured that test kits were available and new staff were hired to ease the VCT workload.

Furthermore, one DMOH explained how increased review and use of data had resulted in improved demand for additional data. He expanded on this by stating that sometimes the DHP tool identifies a problem that requires looking at data not included in the DHP in order to find the answer.

### Barriers to using the DHP tool

In-depth interview participants were also asked about barriers to the use of the DHP tool. Three individuals (one DMOH and two DASCOs) who had been trained but had not used the tool, reported that they were not using the tool because they needed further training, they lacked support from supervisors to use the tool, and they had conflicting priorities. One DASCO explained that the tool was complicated to interpret, while another reported that he was not using the tool because data collection was not part of his role.

Lack of infrastructure, such as computers and printers, was a common barrier cited by all three non-users, as well as by several users. Other tool users reported challenges with saving manually entered data and exporting data.

The fact that Kenya was going through a process of re-districting also affected the use of the tool. New districts that had not been programmed into the tool dropdown menu led to some data discrepancies and analysis issues. Finally, several officials stressed the underlying lack of value placed on data. One respondent explained that if the value of data for program improvement were better understood, the DHP tool would be more widely used.

## Discussion

Results of the in-depth interviews suggest that the DHP tool facilitated data review and decision making among those who used it. Among the users, the findings showed that, although the DHP tool was launched only six months earlier, the tool had a positive effect on data analysis, review, interpretation, sharing, and use at the district level.

The DHP tool was designed to address several of the common barriers to data-informed decision making experienced at the district level in Kenya, including the fragmentation of data reporting, the proliferation of indicators, poor data quality, insufficient data feedback, data feedback in formats that are difficult to understand, insufficient review and interpretation of data, and insufficient use of data to monitor and improve programs. Many of the benefits and experiences cited by tool users suggest that the DHP tool was successful in addressing these barriers. Overwhelmingly, the district-level health officers supported the usefulness of the DHP tool because it improved work efficiency by making data aggregation and analytical work easier. A unique strength of the DHP tool is that it brings together data from multiple sources, which increases availability of data and saves data users significant time that would have been spent retrieving and merging different data sets. Additionally, by combining district data with population-level data, the DHP tool allows data users to compare indicators against population-level denominators, providing more robust and meaningful indicators for analysis and monitoring program trends. The automatic graph and report generators provided visual displays, which aided users in pinpointing problem areas, tracking and analyzing trends, and sharing results with other key stakeholders. The DHP tool provides users with a set of tools to aid them in analyzing data and monitoring program performance.

In addition, through its focus on specific program questions, the tool allowed users to hone in on a key set of indicators and understand not just the change in that indicator over time, but also if that indicator was on track to meet pre-set targets. Additionally, the tool improved data quality by prompting users to personally review data and investigate extreme highs and lows. All of these benefits were reported to enhance use of data in decision making. The tool was also credited for diagnosis of program problems and for improving the ability to have data in-hand to advocate for action around program needs identified in the DHP tool reports. District health officials described using data to inform decision making by addressing problems and developing targeted responses to overcome problems. Informants also provided several anecdotes of specific ways in which they had used the tool to implement successful programmatic changes.

Trained non-users reported that the biggest constraint to using the DHP tool was the lack of computers and other office necessities like printers, printing paper, and even office space. Although they were non-users, it was clear that they were aware of the tool and were often consumers of its results, particularly during quarterly review meetings or other monthly meetings. DHP tool users reported similar barriers including infrastructure and resource requirements. Several users also struggled with some operational aspects of the DHP tool and requested additional training for themselves, as well as some of their colleagues, suggesting that refresher training would be useful. These issues will need to be addressed in order to ensure full access to and more widespread use of the tool. Furthermore, the tool will need to be updated with new districts and relevant district denominator data when district boundaries are revised and new denominator data become available.

Finally, several users stressed a need for broader acceptance of the tool and greater value placed on data. One DHRIO pointed out that data has to be appreciated in order to embrace its usefulness. Improving attitudes towards data and data use will be essential towards improving the acceptance of the tool and its ultimate impact on data quality, data-informed decision making, and the overall functioning of Kenya’s health information system. Foreit et al. [30] posit that regular review and use of data engenders future demand for data; we also observed this phenomenon during the DHP tool training sessions. When trainees were discussing the source of the denominator data, they were unhappy that some were proxy numbers. They discussed the need for more accurate data and called for the collection of such data to improve the accuracy of their district-level decision making. Thus, the authors suggest that the use of the DHP tool may result in a deeper understanding of the value of data in decision making and in turn result in improved attitudes about the usefulness of data in general. Improved attitudes around data at the district level could in turn lead to improved advocacy for additional decision-support tools and improve data use at the sub-national level of the Kenyan health system.

While the assessment showed continued use of the DHP tool among those interviewed at six months post-training, the magnitude of ongoing use is unknown due to lack of resources for continued follow-up. Of note is that the DHP tool was being rolled out at the same time the DHIS was being finalized. The NACC noted the possible overlap of the decision-support functions of the DHP tool and the DHIS, as the DHIS includes a robust decision-support tool that has similar functionalities as the DHP tool. However, it was also noted that the DHP tool would most likely function as a stopgap for decision-support as the DHIS rolled out. Complete reporting into the DHIS could take up to two years and during that time districts would suffer from incomplete reporting into the DHIS. Although district reporting increases each quarter, the DHIS decision-support tools will not be useful until data completeness increases. For example, according to DHIS performance reports, district reporting on PMTCT indicators (using data collection form MOH 731-2) was 28.8% for the quarter of January–March 2012 but increased to 77.1% for the same quarter in 2013 [31]. It is possible that once completeness rates increase, the DHIS will eventually replace the DHP tool. It is also possible that the analyses included in the DHP tool will complement the DHIS and continue alongside the DHIS. While the future is unknown, the fact that the DHP tool proved to be valuable to decision makers even in the presence of the DHIS underscores the fact that there continues to be a need for targeted tools to support decision making as health management information systems are being strengthened.

## Conclusions

This paper stresses the central role that a targeted decision-support tool can play in facilitating the use of information in health decision making at the decentralized level of a health system. The DHP tool is unique because it focuses on programmatic questions and not on a long list of independent indicators, thereby meeting the specific information needs of district-level decision makers. The limiting of the tool’s scope through the inclusion of only 11 questions allows decision makers to target their review of data and quickly access data from multiple fragmented data systems. This also facilitates data review over self-selected time periods and the ability to better understand the status of health programs. This experience has not only improved data-informed decision making but also provides an important experience of how to identify and meet information needs that can be applied to the development of the decision-support tools that will accompany the DHIS.

In addition, the fact that the DHP tool uses technology that is already available in most of the districts is important. DHMTs were familiar with and were already using Microsoft Excel for reporting, which translated into minimal training requirements for users. This is relevant to future rollout or replication in other settings because the training can be added onto existing district-level training opportunities at a low cost. The Microsoft Excel platform also allows for updating the tool (adding districts or questions) to be at relatively low cost because this can be done by experienced users. The experience in Kenya can also be applied to other countries that face challenges making district-level, data-informed decisions with data from fragmented systems.

The small sample size for the qualitative assessment was a weakness of the assessment; more rigorous and longer-term evaluations of targeted decision-support tools are needed. Their contribution to facilitating the review and use of data as well as creating additional demand for data is an important avenue of additional research. Nonetheless, the Kenya experience contributes to the global understanding of how to facilitate the use of data at the district level. The DHP tool showed that a simple, low-tech option can fill an important gap in a transitioning HIS.

## Endnote

^a^HIS are comprised of health information system resources, indicators, data sources, data management, information products, and dissemination and use.

## Abbreviations

DASCO: District AIDS and STI control officer; DHIS: District health information system; DHMT: District health management teams; DHP: District health profile; DHIRO: District health information and records officer; DMOH: District medical officer of health; HIS: Health information systems; MOH: Ministry of health; NASCOP: National AIDS and STD control program; PMTCT: Prevention of mother-to-child transmission; VCT: Voluntary counseling and testing.

## Competing interests

The authors declare that they have no competing interests.

## Authors’ contributions

TN designed the District Health Profile tool, the assessment methodology and data collection tools, and led the data analysis and the development of the manuscript. SM conducted the literature review and contributed to the manuscript. SS supported the design, implementation, and assessment of the DHP tool in Kenya and contributed to the manuscript. All authors read and approved the final manuscript.

## Authors’ information

TN is a senior technical specialist for data demand and use at Futures Group. SM is a research and dissemination associate at Futures Group. SS is a senior technical specialist for monitoring and evaluation at ICF International.
